# PCSK9 Inhibitor Wars: How Does Inclisiran Fit in with Current Monoclonal Antibody Inhibitor Therapy? Considerations for Patient Selection

**DOI:** 10.1007/s11886-022-01782-6

**Published:** 2022-09-10

**Authors:** Natalie Arnold, Wolfgang Koenig

**Affiliations:** 1 Department of Cardiology, University Heart and Vascular Center Hamburg, Hamburg, Germany; 2 German Center for Cardiovascular Research (DZHK), partner site Hamburg/Kiel/Luebeck, Hamburg, Germany; 3 German Heart Center, Munich, Technical University of Munich, Munich, Germany; 4 German Centre for Cardiovascular Research (DZHK), Partner Site Munich Heart Alliance, Munich, Germany; 5 Institute of Epidemiology and Medical Biometry, University of Ulm, Ulm, Germany

**Keywords:** Dyslipidemia, PCSK9 mAb, Evolocumab, Alirocumab, Small interfering RNA, Inclisiran

## Abstract

**Purpose of Review:**

Treatment of dyslipidemia represents one of the most crucial strategies to reduce risk of atherosclerotic cardiovascular (CV) disease (ASCVD). In this review, we critically summarize our knowledge on emerging cholesterol-lowering therapy, targeting PCSK9, paying particular attention on treatment allocation of two drug groups, currently available for clinical use, namely, anti-PCSK9 monoclonal antibodies (mAbs) and inclisiran, a first-in-class small interfering RNA against PCSK9.

**Recent Findings:**

Although both drug classes show a pronounced, but fairly similar reduction in LDL-cholesterol, their long-term safety is still unknown. Compared to mAbs, inclisiran has a more favorable dosing regimen with biannual application that might improve therapeutic adherence significantly. However, a CV outcome trial (CVOT) for inclisiran is still missing.

**Summary:**

If inclisiran will be safe and effective in ongoing/future CVOTs, it has a huge potential to overcome medication non-compliance, thereby providing a powerful therapeutic option to decrease the burden of ASCVD.

## Introduction

Causal involvement of elevated LDL-cholesterol (LDL-C) in the development of atherosclerotic cardiovascular (CV) disease (ASCVD) is undeniable [1, 2]. There is a considerable body of evidence from basic research, epidemiologic, and genetic studies, showing a clear association between long-term exposure to elevated LDL-C and risk of ASCVD [2]. Most importantly, LDL-C remains the main target in ASCVD prevention, and has been shown to reduce CV risk by ~ 20% by each 1 mmol/L (38.7 mg/dL) decrease of the LDL-C level [3].

Since the introduction of the first inhibitor of 3-hydroxy-3-methyglutaryl-coenzyme A reductase, lovastatin, in 1987, statins have been considered the cornerstone of lipid-lowering therapy (LLT). Despite unequivocal evidence of their benefit and long-standing clinical experience concerning safety and tolerability, the “real-world” situation remains disappointing, since only about 20% of patients at very high/high risk achieve the recommended risk-defined LDL-C threshold [4•, 5]. Thus, several representative surveys like the daVINCI or EUROASPIRE [4•, 5] have shown that statins are largely underused and underdosed. In addition, persistence and adherence to statin therapy still represent a key issue for the non-attainment to LDL-C goals. More recently, a retrospective longitudinal analysis of representative prescription data from Germany, including 865,732 patients with newly prescribed statins and 34,490 patients in whom ezetimibe was initiated between July and December 2017, has demonstrated that after 36 months, only 20.6% and 22.3% of subjects, respectively, were still on drug [6••]. Importantly, most patients discontinued LLT within the first 300 days after prescription and did not initiate any other LLT after discontinuation. Therefore, there is still a significant unmet medical need to improve LDL-C management. The identification of proprotein convertase subtilisin-kexin type 9 (PCSK9) opened the possibility for the development of a new class of potent lipid-directed therapeutics for optimal control of LDL-C.

In this narrative review, we focus on current strategies targeting PCSK9 and critically discuss some uncertainties regarding the allocation strategy, paying particular attention on the role of inclisiran in the treatment of dyslipidemia in high-/very high-risk subjects.

## Proprotein Convertase Subtilisin-Kexin Type 9 in Lipid Disorders

**Fig. 1:**
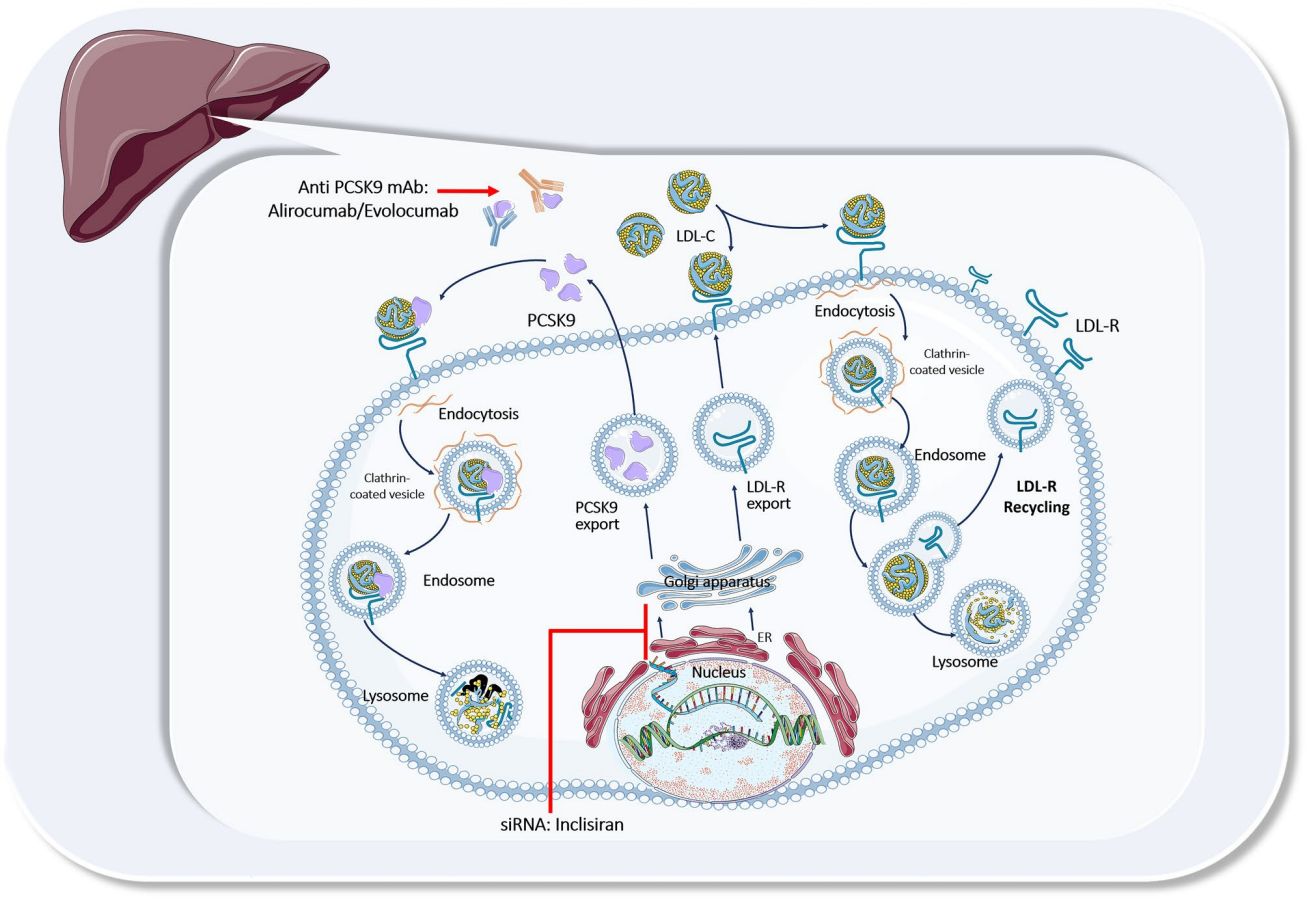
PCSK9-targeted approach for prevention of atherosclerotic cardiovascular disease. A short graphical overview of the role of PCSK9 in lipoprotein metabolism (detailed description is provided in the maint text) and potential targets for marketed PCSK9 inhibitors. PCSK9, proprotein convertase subtilisin-kexin type 9; LDL-R, low-density protein receptor; siRNA, small interfering ribonucleic acid; mAbs, monoclonal antibodies; ER, endoplasmatic reticulum. (Source: The icons in this image are reproduced from Servier Medical Art by Servier. Reproduced from Servier Medical Art by Servier under a Creative Commons [CC BY 4.0] licence.)

PCSK9 represents a classic example of the genetic discovery of a circulating protein, involved in the regulation of atherogenic lipoproteins, thereby revolutionizing our understanding of lipid metabolism. In 2003, a novel gain-of-function (GOF) mutation within the *PCSK9* gene, contributing to a phenotype with markedly elevated LDL-C levels and premature ASCVD, has been identified in patients with severe hypercholesterolemia [7]. A few years later, a loss-of-function (LOF) *PCSK9* mutation has been found to lower PCSK9 activity [8, 9] with a markedly decreased LDL-C concentration and protection from ASCVD. Subsequent studies have demonstrated that modulation of the *PCSK9* gene resulted in a decreased (in case of GOF mutation) or an increased (in case of LOF mutation) number of LDL receptors (LDL-Rs) on the surface of hepatocytes [10, 11], thereby establishing a prominent role of PCSK9 as a key regulator of LDL-C clearance (Fig. 1).

Indeed, the LDL-R pathway represents the primary pathway of LDL-C removal from the systemic circulation. After binding to a LDL-R on the liver surface, the LDL-C/LDL-R complex is internalized by clathrin-mediated endocytosis that results in further LDL-C digestion and processing. Afterwards, the LDL-R is recycled back to the hepatocyte membrane for further uptake and clearance of LDL-C (Fig. 1) [12, 13].

PCSK9, being a serine protease and synthesized mainly in the liver as a precursor, is able to bind to a specific domain of the LDL-R, known as the epidermal growth factor precursor homology domain A (EGF-A) [14]. Such binding triggers endosomal and lysosomal degradation of the LDL-R, subsequently resulting in the prevention of LDL-R recycling that, in turn, leads to decreased LDL-R density on hepatocytes, an inability to attach LDL-C and therefore to a significant reduction of removal of circulating LDL-C (Fig. 1) [12, 13]. Therefore, it is not unexpected that therapeutic inhibition of PCSK9 function became an attractive treatment strategy to lower circulating LDL-C level substantially, as discussed in more detail later.

## PCSK9 Targeted Approach for ASCVD Prevention: Mechanism of Action and Clinical Evidence

Assuming a pivotal pathophysiological role of PCSK9 in lipid regulation, a rapid translation from “bench to bedside” has led to the development of numerous approaches for the successful targeting of PCSK9, including anti-PCSK9 monoclonal antibodies (mAbs), small interfering RNA (siRNA), antisense oligonucleotides (ASOs), anti-PCSK9 small binding proteins (adnectins), vaccines against PCSK9 or CRISPR-Cas9-based *PCSK9* gene editing. Since a variety of comprehensive reviews on novel lipid-lowering agents, directed against PCSK9, have been published within the last few years, we will touch upon each modality only briefly and refer to the most recent publications for more details [15–17].

### Monoclonal Antibodies

The earliest strategy to inhibit PCSK9 was the use of mAbs, selectively targeting PCSK9 receptors, which prevents its binding to the LDL-R. Out of several anti-PCSK9 mAbs (alirocumab (SAR236553/REGN727), evolocumab (AMG145), RG7652, LGT209 (NCT01979601, NCT01859455), 1B20 and bococizumab [18–21], only two of them—the fully human mAbs alirocumab and evolocumab—have been approved for clinical use to date. The research program for bococizumab, a chimeric Ab having 3% murine sequences in the humanized immunoglobulin G antibody, has been abandoned during late stage of development because of a relatively high incidence of anti-bococizumab Abs [21]. Alirocumab and evolocumab, both administrated subcutaneously (s.c.) biweekly or monthly, lower LDL-C by 45 to 60% depending on the dose and application regimen without any major safety issue. The safe and effective reduction in LDL-C resulted in the approvals of both compounds by the Food and Drug Administration (FDA) and the European Medicines Agency (EMA) in 2015, even before any cardiovascular outcome trials (CVOTs) had been published. Two years later, large CVOTs for both evolocumab (FOURIER (Further Cardiovascular Outcomes Research with PCSK9 Inhibition in Subjects with Elevated Risk) [22]) and alirocumab (ODYSSEY OUTCOMES (Evaluation of Cardiovascular Outcomes After an Acute Coronary Syndrome During Treatment with Alirocumab) [23]) have clearly demonstrated a 50–60% reduction in LDL-C concentration during therapy with a mAb (on top of maximal tolerated statin therapy). This has translated into an approximately 15% reduction of future CV risk over a mean follow-up of 2.3 or 2.8 years [22, 23]. These CVOTs have been further confirmed by a meta-analysis of 66,478 patients, who participated in 39 randomized controlled trials with almost identical CV risk reduction of about 15–20% [24]. No increased risk of neurocognitive adverse events, liver enzymes elevations, rhabdomyolysis, or new-onset diabetes mellitus could be seen. Furthermore, a first indirect comparison between alirocumab and evolocumab [25••], again using a meta-analysis strategy (30 trials with 59,026 patients), has been undertaken and has demonstrated a comparable efficacy of both agents in reducing risk of future ASCVD events. In addition, we have now evidence that evolocumab and alirocumab treatment, on top of statins, in patients with acute coronary syndrome (ACS) modifies coronary plaque properties, leading not only to a significant thickening of the fibrous cap, thereby stabilizing it, but also resulting in regression of atheroma volume, as has been recently shown within the HUYGENS (High-Resolution Assessment of Coronary Plaques in a Global Evolocumab Randomized Study) [26•] and the PACMAN-AMI (Effects of the PCSK9 Antibody Alirocumab on Coronary Atherosclerosis in Patients With Acute Myocardial Infarction) [27•] studies. Nevertheless, despite strong beneficial effects of these compounds on top of maximum tolerated statin/ezetimibe therapy, there are still some aspects that hamper the wide implementation of evolocumab/alirocumab in clinical routine. One of them relates to their still fairly high costs and the consecutive restrictions by health care providers regarding reimbursement and subsequent difficulties in the prescription procedure, since prior authorization requirements for PCSK9 inhibitors seem to be more extensive as compared with other cardiometabolic drugs [28]. Although several attempts for the broader implementation of both anti-PCSK9 mAbs have been already undertaken, such as, e.g., cutting the price for both drugs by approximately 60% as in the USA, nevertheless, a practical near-term approach is still highly appreciated for better initiation of such therapy in clinical routine.

### Small Interfering RNA (siRNA)

Another compound targeting PCSK9, already used in the clinical routine, is inclisiran, a first-in-class cholesterol-lowering small interfering ribonucleic acid (siRNA) [29]. Inclisiran has a double-stranded structure and is composed of 21–23 nucleotide sequences. In contrast to anti-PCSK9 mAbs, it inactivates PCSK9 by inhibition of its hepatic synthesis. Its highly selective delivery to hepatocytes results from the fact that the sense strand of inclisiran is conjugated with triantennary *N*-acetylgalactosamine (GalNAc), which interacts with asialoglycoprotein receptors (ASGPRs) on the liver surface, and thereby facilitating the direct uptake of inclisiran by hepatocytes. After entering the hepatocyte, the guide/antisense strand of inclisiran binds to the RNA-induced silencing complex (RISC) of *PCSK9*, thus preventing the intracellular translation of PCSK9 mRNA to protein. This, in turn, increases LDL-R recycling on the hepatocyte surface and subsequent increase of LDL-C uptake and decrease of LDL-C levels in the circulation. The detailed mechanism of action of inclisiran has been reported previously elsewhere [30–33].

The safety, tolerability, and efficacy of inclisiran have been studied in detail within the ORION clinical development program [29, 34–38, 39••], which includes several completed and still ongoing trials (details of these trials have been described in several recent reviews) [31–33].

In brief, the data derived from the phase I and II trials led to a discovery of an optimal administration regimen for inclisiran—s.c. injection of 284 mg (equivalent to 300 mg inclisiran sodium) at 0–90–180 days and every 6 months thereafter. More recently, a pooled patient-level meta-analysis from the phase 3 ORION program (ORION-9, ORION-10, and ORION-11) including 3660 subjects with familial hypercholesterolaemia (FH), established ASCVD or those in the high-risk primary prevention category (ASCVD risk equivalents or heterozygous FH (HeFH)) demonstrated a sustained PCSK9 suppression of about 80% and time-adjusted LDL-C reduction of approximately 50% from baseline [39••]. Furthermore, the safety profile of inclisiran was found to be comparable to that of anti-PCSK9 mAbs with no major serious adverse events. The most commonly reported adverse effect was a mild to moderate, but transient injection site reaction, which led to the discontinuation of the drug in only a very small percentage of patients. Yet, the first inclisiran CVOT—ORION-4—is still ongoing and estimated to be completed in 2026 (based on the information from ClinicalTrial.gov) [40••]. ORION-4, which has recruited patients only in the UK and the USA, will be followed by a second CVOT, VICTORION-2 PREVENT which will be carried out worldwide [41]. Despite still ongoing CVOTs, inclisiran has already been approved by EMA in 2020 and by FDA in 2021 for the treatment of hypercholesterolemia or mixed dyslipidemia.

### Emerging PCSK9 Inhibitors

To date, there are also several next-generation PCSK9 inhibitors in the pipeline, including LIB003 (Lerodalcibep), AZD8233 (ION449), CIVI-008 (a third-generation PCSK9 antisense molecule, called cepadacursen sodium), or MK-0616, which might be more cost-effective than currently available drugs. Lerodalcibep (LIB003) represents a recombinant fusion protein, consisting of a PCSK9-binding domain (adnectin) conjugated with human albumin, that enables a half-life time of 12 to 15 days [16]. Acting through the binding to PCSK9 and thus preventing an interaction with the LDL-R, lerodalcibep in a dose of 300 mg s.c. once per month is able to reduce LDL-C concentration by 77% from baseline to 12 weeks of treatment [42] and by a mean of ~ 60% over a 36-week dosing period [43]. Currently, LIB003 is being evaluated in clinical phase III trials [44].

More recently, positive results from the ETESIAN Phase IIb trial, investigating a first chemically modified, GalNAc-linked 16-mer ASO to target PCSK9 gene expression in the nucleus (AZD8233 also known as ION449 [45]), have been reported, showing a 73% reduction in LDL-C in a dose of 50 mg from baseline to week 12 [46].

Intriguingly, data on the efficacy and safety of the first orally bioavailable PCSK9 inhibitor in early stage development have been presented recently [47]. Oral tricyclic macrocycle MK-0616, as a synthetic cyclic peptide that inhibits PCSK9, besides absence of any serious side effects demonstrated an almost 90% reduction in circulating PCSK9 concentrations and may have even a slightly more potent LDL-C-lowering capacity of about 65% compared to s.c. administrated of anti-PCSK9-mAbs in phase I clinical trials [46]. MK-0616 is currently in phase II clinical developmental [48].

Other strategies, such as CRISPR-Cas gene editing and PCSK9 vaccine, are only in preclinical studies or phase I clinical trials [49, 50].

Thus, the pharmacologic armamentarium to lower LDL-C by targeting PCSK9 has been expanded drastically during recent years. Table 1 provides a short overview of already available PCSK9 inhibitors or those being in the developmental process.

**Table 1:** Established and novel strategies of PCSK9 inhibition

Agent	Description	Mechanism of action	Administration	~ LDL reduction	Latest stage of development
**Evolocumab (Repatha®)**	Humanized monoclonal antibody	Extracellular binding to catalytic domain of circulationg PCSK9, with further prevention of PCSK9/LDL-R interaction and subsequent increase in LDL-R recycling	140 mg s.c. biweekly	~ 60%	Approved
			420 mg s.c. monthly	~ 55%	
**Alirocumab (Praluent®)**	Humanized monoclonal antibody	Extracellular binding to catalytic domain of circulationg PCSK9, with further prevention of PCSK9/LDL-R interaction and subsequent increase in LDL-R recycling	75 mg s.c. biweekly	~ 45%	Approved
			150 mg s.c. biweekly	~ 60%	
			300 mg s.c. monthly	~ 50%	
**Inclisiran (Leqvio®)**	Double-stranded, GalNAc-conjugated small interfering RNA	Intrahepatic binding to RISC of PCSK9 with further inhibition of PCSK9 protein synthesis	284 mg s.c. biannually (after first additional boostering)	~ 50%	Approved
**Lerodalcibep (LIB003)**	Recombinant fusion protein, consisting of PCSK9-binding domain (adnectin), conjugated with HSA	Extracellular binding and neutralization of PCSK9, with further prevention of PCSK9/LDL-R interaction and subsequent increase in LDL-R recycling	300 mg s.c. monthly	~ 60– ~ 77%	Phase III
**AZD8233**	GalNAc-conjugated 16-nucleotide antisense oligonucleotide	Intrahepatic degradation of PCSK9 mRNA with further inhibition of PCSK9 protein production	50 mg s.c. monthly	~ 73%	Phase II
**MK-0616**	Synthetic tricyclic peptide, targeting PCSK9	Binding to the flat PCSK9:LDL-R interface with mAb-like affinity, thereby preventing thier interaction	10–20 mg oral once daily	~ 65%	Phase II
**Cepadacursen sodium**	Third-generation LNA antisense oligonucleotides	Suppression of PCSK9 expression	CIVI-007 (75 mg) s.c monthly	~ 60%	Phase II
			CIVI-008 oral	-	Preclinical
**NNC0385-0434**	PCSK9 inhibiting peptide	Not provided	Oral, once daily	-	Phase II
**CVI-LM001**	First-in-class small molecule PCSK9 inhibitor	Dual mechanism of action: inhibition of PCSK9 transcription and prevention of LDL-R mRNA degradation	Oral, once daily	~ 25%	Phase II
**PCSK9 peptide Vaccine (AT04A and AT06A)**	Short peptide mimicking the N-terminal domain of PCSK9	Eliciting production of autoantibodies against PCSK9	Priming immunizations at weeks 0, 4, and 8, and one booster immunization at week 60 s.c	~ 50%	Phase I
**CRISPR-Cas9**	PCSK9 gene editing	Knockout/knockdown of PCSK9 expression	Once-and-done therapies	~ 60%	Preclinical

*PCSK9* proprotein convertase subtilisin-kexin type 9, *LDL-R* low-density protein receptor, *GalNAc N*-acetylgalactosamine, *RNA* ribonucleic acid, *RISC* RNA-induced silencing complex, *HAS* human serum albumin, *mAbs* monoclonal antibodies, *LNA* locked nucleic acid, *CRISPR* Clustered Regularly Interspaced Short Palindromic Repeats

## PCSK9 Inhibition: Critical Issues and Appropriate Therapy Allocation

Taken together, there is strong genetic, epidemiological, and clinical proof-of-concept data that targeting PCSK9 significantly reduces plasma LDL-C levels and lowers the risk of ASCVD at least for the mAbs, as discussed above. As already mentioned, to date, only two groups of PCSK9 inhibitors—mAbs (evolocumab [Repatha®] or alirocumab [Praluent®]) and one siRNA (inclisiran [Leqvio®])—are available in clinical practice. Although it seems to be clear that subjects at very high and highest risk represent the target population, which would benefit most from both lipid-lowering strategies, we are still dealing with a significant clinical dilemma, since a major practical question remains still unanswered—how to decide which patient would be the best candidate to be treated with PCSK9 mAbs and which with inclisiran? Which criteria makes a patient suitable for which agent? Unfortunately, until now, there is no conclusive evidence due to lack of head-to-head trials between inclisiran and anti-PCSK9 mAbs, making a choice of appropriate therapy allocation challenging. Nonetheless, there are several points, such as, e.g., baseline LDL-C levels and desirable/necessary LDL-C reduction, adherence to therapy and application in the primary (in the future) or secondary prevention setting, which might facilitate decision making in such a patient-tailored approach.

### Comparable Effect on LDL-C Reduction

When added to statins and ezetimibe, both therapeutic groups (mAbs and siRNA) demonstrate a comparable LDL-C-lowering potential. Alirocumab in a biweekly administered regimen is able to lower LDL-C by 45 to 60% depending on the applied dose (75 mg vs 150 mg) and by ~ 50% while given monthly in a 300-mg dose. Evolocumab is currently the most effective of the PCSK9 inhibitors so far [51] and reduces LDL-C during biweekly dosing of 140 mg by approximately 60%, whereas an application of 420 mg once per month (either by three 1 mL auto-injectors or by Pushtronix® infusion device) results in a LDL-C lowering of ~ 55%. Inclisiran reduced LDL-C by approximately 50% at peak effect (30 days post-dose), and at day 180, LDL-C levels were still reduced by approximately 53%.

### Variability of Drug Response

One point to be noted here, however, is the inter-individual variability seen for anti-PCSK9 mAbs as well as for inclisiran. Of interest, CVOTs for PCSK9-inhibiting mAbs [22, 23, 52–54] as well as a pooled ORION analysis [39••] revealed a small proportion of participants as non- or hypo-responders (< 15% LDL-C reduction). Whether real-world data would provide more pronounced differences regarding inter-individual variability, different from those reported within the CVOTs/phase III trials, still has to be elucidated. More recently, first, preliminary data from the German Inclisiran Network, including 117 patients on inclisiran therapy, have been presented, demonstrating a fairly high inter-individual variability of LDL-C lowering 3 months later after the first inclisiran application [55]. Therefore, more research in larger patient populations is urgently needed to answer several still critical questions.

### The Crucial Problem: Adherence to Drug Therapy

It has already become evident that adherence and persistence to PCSK9-inhibiting mAb therapy remains a significant concern, since it seems to be much lower in clinical routine than in CVOTs. Thus, it has been shown that 30 to 40% of patients discontinued their anti-PCSK9 mAb therapy during/after 6 months of treatment initiation [56, 57]. More importantly, a current in-depth analysis of real-world adherence and persistence data to LLT in Germany revealed that among 1940 newly prescribed anti-PCSK9 mAb users, only half of them remained on treatment after 36 months [6••]. Although existing difficulties in reimbursement might at least partially explain the poor adherence/persistence to anti-PCSK9 mAb therapy, overall compliance to alirocumab/evolocumab still represents a matter of concern.

In this regard, implementation of inclisiran into the LDL-C-lowering armamentarium might represent a cutting edge for current cholesterol management by potentially significantly improving long-term adherence to LLT. As discussed earlier, data from the ORION program have impressively shown a sustained suppression of both PCSK9 and LDL-C for at least 6 months, thereby allowing to administer inclisiran biannually, whereas anti-PCSK9 mAbs require 12–26 injections per year. More importantly, its twice-yearly administration with sustainable LDL-C lowering of about 50% on top of maximally tolerated statin/ezetimibe therapy might theoretically lead to an almost complete resolution of non-adherence, since inclisiran is intended to be administrated by healthcare professionals. This, in addition, would provide the advantage that the patient is under tight control by his physician. By contrast, anti-PCSK9 mAbs are self-applicable and require adequate refrigeration; therefore, inappropriate injection/storage cannot not be excluded completely. Indeed, the most frequent cause of resistance to anti-PCSK9 mAbs is their impaired entry into the systemic circulation due to poor adherence and improper administration technique [58•, 59]. The development of anti-drug neutralizing Abs against evolocumab or alirocumab, however, can be considered negligible (e.g., < 2% in FOURIER) [22] and may not significantly affect drug efficacy.

Thus, an approach, with an injection burden of twice yearly (after the first year where an additional booster injection is needed) using pre-filled, single-use 1.5-mL syringes seems to be very promising. On the other hand, all so far conducted studies were undertaken on top of statin therapy (with or without ezetimibe), meaning that most of the patients would still take statin pills 365 days per year in addition to PCSK9 inhibition therapy. Whether inclisiran would have the same magnitude of LDL-C lowering without concomitant statin therapy and especially their beneficial pleoptopic effects is not completely clear and probably would be difficult to explore, since conduction of such trials is definitely ethically inappropriate and might be feasible only in statin-intolerant patients. Subgroup analysis from the pooled patient-level analysis of ORION 9–11 studies demonstrated no significant differences in LDL-C reduction under inclisiran treatment in those with or without statin therapy at baseline, with a least squares mean percent difference in LDL-C of – 54.5 versus – 48.8, respectively [39••]. Furthermore, although durable LDL-C reduction represents a major advantage of inclisiran therapy, it might at the same time be potentially associated with its major disadvantage, if adverse effects would be experienced. This fact might therefore have a significant impact on the overall patients’ perception/acceptability of the drug.

More importantly, it still remains unknown whether LDL-C lowering by inclisiran will translate into similar reductions of CV events. Unfortunately, we already know that significant LDL-C lowering not always results in appropriate CV risk reduction due to competing residual risks [60]. However, targeting the specific, well-validated PCSK9 pathway has been shown promising, and most likely we will also see a significant reduction in CV events in inclisiran CVOTs comparable to those from trials with mABs. Clear evidence to support this notion comes from both genetic studies, where subjects with a LOF *PCSK9* mutation do not have any apparent secondary adverse effect or other metabolic disturbances, but demonstrate a lower risk for ASCVD [61, 62], as well as from CVOT for evolocumab and alirocumab [22, 23], where CV risk reduction under treatment was found to be 15%. The intriguing point remains whether inclisiran would provide an even stronger benefit on CV morbidity and mortality, compared to PCSK-9 inhibition by mAbs, due to the fact that in contrast to anti-PCSK9 mAbs, which are directed against circulating PCSK9, inclisiran inhibits PCSK9 synthesis intracellularly, thereby potentially having additional effects beyond modulation of LDL-R expression. Indeed, PCSK9 has a variety of pleiotropic effects, including involvement in inflammatory pathways or thrombogenesis [63]. On the other hand, theoretically, one could speculate that mAbs lead to a more complete inactivation of PCSK9, since they bind to all circulating PCSK9, including that generated by other organs like, e.g., intestine, endocrine pancreas, or the brain [64•], whereas inclisiran only inhibits intracellular hepatic biosynthesis of PCSK9. Interestingly, to date, several attempts have been already undertaken to assess rates of CV events under inclisiran therapy using available data from ORION trials, where CV risk reduction was ranging from a 24% decrease in MACE (CV death, cardiac arrest, non-fatal myocardial infarction, or non-fatal stroke) compared to placebo [65] to only marginal [66] or no risk reduction at all [67]. However, all these trials were underpowered, and the result on ORION-4 CVOT, including approximately 15,000 participants with a follow-up of 5 years, [39••], has to be awaited before meaningful conclusions with regards to CV risk reduction can be drawn.

Since inclisiran targets PCSK9 intrahepatically, it might be also a promising option [68] in the treatment of receptor-negative variants of homozygous FH (HoFH) (i.e., completely inactive LDL-R) [69], a situation, where anti-PCSK9 mAbs would probably only be mildly effective or even fail to lower LDL-C, as it has been already seen in patients with HoFH and LDL-R-negative mutations in both alleles, where no response to evolocumab was observed [70–72].

A final point of inclisiran “attractivity” is again related to its sustainable LDL-C reduction, which might be essential in a primary prevention setting, where, e.g., only moderate LDL-C lowering is required, that can be easily done by only one inclisiran injection per year, since a 29.5 to 38.7% LDL-C reduction (time-averaged) over 1 year is achievable with a single dose of inclisiran [36]. Still, at present, there are no data in the primary prevention setting available and the above notion remains speculative.

Taken together, inclisiran might be used not only as a “second-option” therapy in subjects with a history of poor response or adverse effects to both marketed PCSK9-inhibiting mAbs or in those patients with obvious adherence problems. In contrast, it might even be suitable for a much broader spectrum of implementation due to its appealing twice-yearly dosing schedule, provided, however, it will reduce CV risk significantly, show no severe long-term effects, and be cost-effective.

## Conclusion

The latest European Society of Cardiology/European Atherosclerosis Society (ESC/EAS) 2019 guidelines for dyslipidemia management with revised LDL-C treatment goals (< 55 mg/dL for patients at very high risk) made their achievement with conventional LLT using statins and/or ezetimibe even more difficult [73], implicating that many patients would need more potent lipid-lowering drugs such as PCSK9 inhibitors to achieve these goals.

In the context of an expanding pharmacopeia of therapeutic modalities, directed against PCSK9, the crucial factor, which might probably solve the PCSK9 inhibitor battle, will be most probably related to improved LLT compliance and inclisiran might take a leading role in this context.
